# Functional Connectivity Signatures Underlying Simultaneous Language Translation in Interpreters and Non-Interpreters of Mandarin and English: An fNIRS Study

**DOI:** 10.3390/brainsci12020273

**Published:** 2022-02-16

**Authors:** Yan He, Yinying Hu

**Affiliations:** 1 College of Foreign Languages and Literatures, Fudan University, Shanghai 200433, China; heyanhy@fudan.edu.cn; 2 Institute of Brain and Education Innovation, East China Normal University, 3663 North Zhongshan Road, Shanghai 200062, China

**Keywords:** simultaneous language translation, functional connectivity, fNIRS, right dorsolateral prefrontal cortex, interpreters, non-interpreters

## Abstract

Recent neuroimaging research has suggested that interpreters and non-interpreters elicit different brain activation patterns during simultaneous language translation. However, whether these two groups have different functional connectivity during such a task, and how the neural coupling is among brain subregions, are still not well understood. In this study, we recruited Mandarin (L1)/English (L2) interpreters and non-interpreter bilinguals, whom we asked to perform simultaneous language translation and reading tasks. Functional near-infrared spectroscopy (fNIRS) was used to collect cortical brain data for participants during each task, using 68 channels that covered the prefrontal cortex and the bilateral perisylvian regions. Our findings revealed both interpreter and non-interpreter groups recruited the right dorsolateral prefrontal hub when completing the simultaneous language translation tasks. We also found different functional connectivity between the groups. The interpreter group was characterized by information exchange between the frontal cortex and Wernicke’s area. In comparison, the non-interpreter group revealed neural coupling between the frontal cortex and Broca’s area. These findings indicate expertise modulates functional connectivity, possibly because of more developed cognitive skills associated with executive functions in interpreters.

## 1. Introduction

Exceptional performance is what human beings strive for. People try their utmost to figure out what underpins the myth of excellence. How do experts obtain exceptional performance? How do their brains function? What are the neural mechanisms in the brains of experts, compared with non-experts? To answer these questions, quite a few neuroimaging studies have been conducted to compare brain mechanisms between experts and non-experts, regarding expertise levels in multiple fields, such as mathematics, chess playing, music, sports, etc. [1,2,3,4]. This is also the scenario in the field of simultaneous language translation.

Simultaneous language translation is a highly complex cognitive activity, involving the transfer of one language into another with concurrent language input. It has attracted considerable attention among the neuroimaging community [5,6,7,8,9,10,11,12,13,14,15,16,17,18]. To understand exceptional performance in simultaneous language translation, scholars [7,8,11,13,14,19] compared neural mechanisms between interpreters and non-interpreters. The studies have revealed different brain activation patterns between interpreters and non-interpreters [8] and stronger executive control in interpreters [13,14], suggesting expertise-dependent neural modulations in the brains of interpreters. However, language activities, such as production and comprehension, are supported by a distributed network of cortices, encompassing the language areas, temporal regions, as well as the visual areas [20,21]. Studies have suggested that bilinguals or multilinguals, in particular, rely heavily on the coordination of multiple brain regions, i.e., the executive-control-related area and language areas, to manage language switching, monitoring, inhibitory control, etc. [22]. Yet, compared to the vigorous exploration of the segregated brain activation underlying simultaneous language translation, only a handful of studies have been conducted to investigate how brain sub-regions coordinate to complete such a task, namely the functional connectivity (FC).

Becker et al. [5] found that the left frontal pole was strongly connected to the left inferior frontal gyrus, as well as the middle temporal gyrus, in professional interpreters compared to multilingual controls. However, the study did not employ simultaneous language translation tasks, but tasks only measuring aspects of cognitive control. Elmer and Kühnis [6] identified increased left-hemispheric theta phase synchronization and FC strength in the left dorsal pathway in simultaneous interpreters. Yet, the study did not use simultaneous language translation tasks, either, but auditory semantic decision tasks. The first study exploring FC underlying simultaneous language translation was conducted by García et al. [12], who observed, based on their scalp EEG recordings obtained from ten professional translators, greater information exchange in the right temporo-occipital networks for backward translation (translating from L2 to L1), and in bilateral frontotemporal networks for forward translation (translating from L1 to L2), both compared to the other direction. More recently, Zheng et al. [9] reported increased FC between the left anterior temporal lobe and the right thalamus for backward translation, whilst enhanced FC between the left anterior temporal lobe and the left inferior frontal, left orbitofrontal as well as bilateral parietal clusters for forward translation. These oral-translation-task-based studies explored FC either in professional interpreters or in trainee interpreters. So far, no study has compared the neural substrates of FC underpinning simultaneous language translation in different expertise groups. The lingering question is whether interpreters and non-interpreters have identical FC pattern during simultaneous language translation tasks. To bridge this gap, we conducted the first neuroimaging study addressing the issue. Based on the above-mentioned expertise- or translation-related studies, we hypothesized that functional connectivity underlying simultaneous language translation is also modulated by expertise, namely interpreters and non-interpreters have different FC patterns; and this difference lies in networks connecting cognitive control areas and language areas. To present a larger picture, we investigated not only the difference of FC between groups, but also the FC in each group.

Lesion studies have reported the cortical representation of both Chinese and English processing in Broca’s area, the premotor cortex, Wernicke’s area, as well the parietotemporal boundary [23]. Moreover, neuroimaging studies regarding translation or interpreting, have identified significant activation in inferior and dorsolateral frontal region [8,14,17,23], prefrontal regions [15,16,17,24,25], Broca’s area [25,26,27], as well as the left temporal area [14,18,28] during the task, suggesting the above-mentioned cortical regions are of paramount importance in the cognitive processing of interlingual brokering. These areas were our focus for neural imaging observation. Broca’s area has been proved to play an important role in various language related activities [29]. It coordinates information transformation across large-scale networks of spoken word production [30]. Meanwhile, studies have suggested it to be more involved in the cognitive functions, such as lexical retrieval, cognitive control, and verbal working memory during language processing [31,32]. While for Wernicke’s area, studies have suggested that it supports retrieval of metal representations of phoneme sequences, which are used for short-term memory tasks and speech output [33]. Apart from this, the research conducted by Harpaz et al. [34] suggested that this area plays a role when processing and determining the secondary meaning of ambiguous words.

In addition to traditional language areas, we also investigated the classic brain region related to executive functions, namely the dorsolateral prefrontal cortex (DLPFC), supporting such cognitive processes as planning, inhibition, and abstract reasoning. It is reported to have correlation to task monitoring, especially during unfamiliar or attention demanding tasks [35]. Several bilingual studies have hypothesized a correlation between DLPFC and language switching in the bilinguals, an ability to activate the target language while inhibiting the non-target language [36,37]. Moreover, a previous study [38] had proved that DLPFC is also important for holding several pieces of transitory information in mind for a period of time before the information is processed.

Our areas of interest (AOIs) also included the inferior parietal lobule as research has shown its engagement in language processing. The intraoperative electrocortical study found both English and Chinese production activate the parietotemporal boundary [23]. The study conducted by Hartwigsen et al. [39] found brain activation in bilateral supramarginal gyri during phonological word choice task. Together with the angular gyrus, it helps to map words with meanings. Impairment to this area may also lead to receptive aphasia [40]. While the angular gyrus is revealed to play a role in high-order language activities, such as reading, writing and speaking (ibid). The angular gyrus contributes to obtaining meaning from visually presented words, by transferring the visual information it receives to Wernicke’s area (ibid). Apart from its involvement in language functions, the angular gyrus also credits to memory retrieval, being aware of the gap between what is expected and what is abnormal [41]. To provide a comprehensive view of the neural substrates of FC, the above-mentioned brain regions are our AOIs for investigation.

In summary, the present study investigated whether interpreters and non-interpreters have congruent FC pattern in bilateral inferior gyrus and inferior parietal lobule, as well as the DLPFC during simultaneous language translation tasks. It is worth mentioning that, in our study, we define interpreters as the bilinguals who have at least 12 months of interpreting experience, while non-interpreters as the bilinguals without interpreting experience. fNIRS, which offers unsurpassed temporal resolution and provides quantitative hemodynamic information regarding oxyhemoglobin (HbO) and deoxyhemoglobin (HbR) [42], was employed in the current study. fNIRS has been shown to be relatively insensitive to movement artifacts, allowing study designs to include body movements; this is helpful since the present study requires continuous overt speech production and continuous measurements for several experiment conditions.

## 2. Methods

### 2.1. Participants

A total of 16 interpreters (2 males, mean age = 25.00 ± 2.39 years) took part in this study as the interpreter group (IG), recruited from Shanghai International Studies University. They all passed the Accreditation Test for Translators and Interpreters in China and obtained the advanced level interpreting certificate. A total of 16 postgraduates (4 males, mean age = 23.88 ± 1.59 years) from East China Normal University were recruited as the non-interpreter group (NIG) with age, education, working memory, L2 proficiency, the onset age of L2 acquisition, and the total time length of L2 acquisition matched to the IG (see Table 1 for details). The sample size in the current research was based on previous studies [6,9]. Notably, the working memory span test was used to measure working memory, since a strong correlation between working memory and executive function was revealed in previous studies [43,44]; the subtests of TOEFL (i.e., Reading Comprehension and Structure and Written Expression) were employed to examine L2 proficiency, according to the suggestion given by Hulstijn [45]. Each participant in the NIG had never been exposed to any professional interpreting training prior to the experiment.

All participants were native Mandarin (the standard form of Chinese, which is the official language of China, L1) speakers, with English (L2) as their foreign language. All participants started learning English at elementary school; therefore, they were all unbalanced bilinguals. They were right-handed, with normal or corrected-to-normal vision and had no history of neurological or psychiatric disorders. Each participant completed informed consent forms prior to the experiment and were paid with monetary compensation for their participation. This study was approved by the Committee on Human Research Protection of East China Normal University (HR 094-2018).

### 2.2. Materials

A total of 48 Mandarin and 48 English sentences were used in this study, in accordance with the stimulus format used in previous neuroimaging research on simultaneous language translation [14,46]. All sentences were created with exactly the same structure, which was “subject + verb + object + complement”. By using predicate verbs and infinitive phrases, the general structure of each sentence was “I/he/she/you/we/they + verb + (somebody) + to do (something)” in Mandarin/English. For the Mandarin and English sentences, the word count, word frequency, pronoun density, notional word density, and translatability (the difficulty of sight translating a sentence, as rated by five interpreting teachers) were controlled for subsequent comparative analysis across language (see Table 2).

### 2.3. Procedure

We used sight translation and reading aloud as the experimental tasks. Sight translation is one of interpreting modalities, the transferring of a visual message in one language into an oral message in another language. It requires language switching efforts [47], information retention capacity [48], coordination between comprehension, memory and production efforts [49], which are indispensable cognitive processing in performing interpreting. It has been an essential part in interpreting training. Reading aloud tasks were used as baseline tasks.

The participants were instructed to perform four tasks: (1) sight translation from Mandarin to English (forward translation, FT), (2) sight translation from English to Mandarin (backward translation, BT), (3) reading aloud in Mandarin (M), and (4) reading aloud in English (E). We investigated both translation directions, since a myriad of studies have confirmed that forward and backward translation have different brain activation patterns [8,9,16,24]. Each task included two blocks. The eight blocks were presented in a pseudo-random order, with a rest of 20 s between every two blocks. Each block began with a piece of instruction and ended with a cue to notify the participants the ending of the block. There were 12 trials in each block and, thus, 96 trials in total.

**Figure 1:**
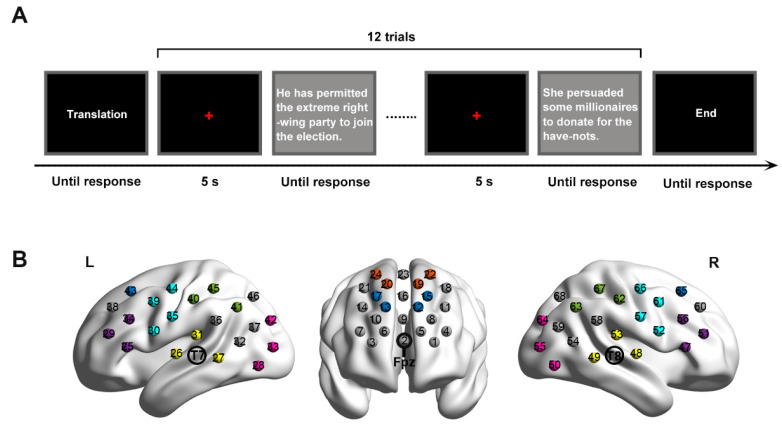
(**A**) One block of experimental procedure. (**B**) fNIRS channel configuration. Shaded different colors show the different brain areas (dark blue: dorsolateral prefrontal cortex. Orange: frontal eye fields. Blue–green: motor cortex. Purple: Broca’s area. Glass green: Wernicke’s area. Yellow: temporal cortex. Pink: visual cortex. L: left. R: right).

Each trial started with a red fixation cross of 5 s, presented in the center of a black screen (Figure 1A). Then, one sentence appeared in a white color at the center of a grey screen. The sentence disappeared until participants responded to it (i.e., reading aloud or sight translation) by pressing a button (i.e., the space bar). A digital voice recorder was used to record participants’ oral outputs.

The experimental tasks were programmed by using the E-prime software 2.0 (Psychology Software Tools, Sharpsburg, PA, USA). Before the formal experiment, participants were given several practices to be familiar with the experimental tasks and procedure.

### 2.4. fNIRS Data Acquisition

The fNIRS data were collected by using an ETG-7100 Optical Topography system (Hitachi Medical Co., Kashiwa, Japan), with two wavelengths of near-infrared light (695 and 830 nm). The distance between optodes was 3 cm. The sampling rate was 10 Hz.

To cover our AOIs, three optode probe patches were adopted, with one 4 × 4 patch and two 3 × 5 patches (Figure 1B). The 4 × 4 patch included 8 emitter probes and 8 detector probes, thus forming 24 channels (channel 1–channel 24). It was placed on the participant’s head such that the middle position in the lowest row of the patch was pointed at the EIG electrode of Fpz. Such position was based on previous findings that the prefrontal cortex was defined as the area above the electrode locations of Fp1–Fp2 [50,51]. The 3 × 5 patches included 8 emitter probes and 7 detector probes each, with 22 recording channels for the right hemisphere (channel 25–channel 47) and 22 channels for the left hemisphere (channel 48–channel 68). They were positioned such that the middle optode in the lowest row of the patch was placed over the EIG electrodes of T7 and T8 on the left and right hemispheres, respectively. The two patches were positioned according to the locations used in previous research, for instance, Broca’s area was defined as the cross point between T7-Fz and F7-Cz; T8-Fz and F8-Cz [34,52]; Wernicke’s area and the temporal cortex were described as the cross point between T7-P3 and C3-P7; T8-P4 and C4-P8 [46,53]. To ensure the accuracy of the placement, the patches were mounted on a homemade fNIRS cap, which was an elastic swimming cap of normal size but aligned to a standard EEG cap with 10–20 configuration.

The brain regions that covered by the fNIRS channels were estimated using a 3D-digitizer (PATRIOT, Polhemus, Colchester, VT, USA) and the NIRS-SPM software (Wellcome Trust Centre for Neuroimaging, London, UK) [54]. Specifically, the 3D-digitizer was used to identify the corresponding positions of channels on the physical heads [55], while the NIRS-SPM software for MATLAB validated the standard brain model and data yielded by the 3D-digitizer [56].

### 2.5. Data Analysis

#### 2.5.1. Behavioral Performance

Participants’ oral outputs during the experimental tasks were assessed by a panel of five interpreting teachers and scholars, including three native Mandarin speakers and two native English speakers. For the sight translation tasks (i.e., FT and BT), the oral outputs of each trial were evaluated on a 10-point scale, with 10 representing the full score, indicating a successful sight translation, and 0 indicating a failed sight translation. The accuracy, fluency, and appropriateness of sight translation were defined as the scoring indexes. Any inaccurate expressions, incomplete information, disfluency, or the use of inappropriate expressions or tones resulted in lower scores. For the reading tasks (i.e., E and M), the oral outputs of each trial were also scored for accuracy and fluency on a 10-point scale, with 10 representing the full score and 0 suggesting a failed reading trail. Any inaccurate pronunciation and disfluency resulted in lower scores.

To compare the behavioral performance among tasks, stimuli, and groups of participants, the rating scores were entered into a 2 × 2 mixed design ANOVA with subject group (IG vs. NIG) as the between subject variable, sight translating/reading direction (FT vs. BT or E vs. C) as the within subject variable.

#### 2.5.2. fNIRS Data Analysis

The fNIRS data were preprocessed via MATLAB 2020a (MathWorks, Natick, MA, USA). The raw data were firstly converted into changes in concentrations of HbO and HbR through the Modified Beer–Lambert Law. In this study, HbO was selected to enter into subsequent analysis since it was sensitive to regional cerebral oxygenation changes and neural activity [57] and correlated with the fMRI signal [58]. After conversion, the principal component spatial filtering method [59] was applied to remove the global systemic noise, with setting the angle of the spatial filter as 46. The correlation-based signal improvement method [60] was subsequently used to remove head motion artifacts. This method is based on the observation that HbO and HbR values are usually negatively correlated, but that head motion induces a positive correlation.

To account for possible differences in optode localization due to different head sizes and shapes of participants, the preprocessed data from 42 channels were averaged for our AOIs before further analysis. The 42 channels that shared a common fNIRS source were averaged together resulting in eight brain regions centered on source locations, including: dorsolateral prefrontal cortex (DLPFC), frontal eye fields (FEF), motor cortex (MC), Broca’s area (BA), Wernicke’s area (WA), temporal cortex (TC), and visual cortex (VC). Considering the left and right hemispheres, the 7 regions were then divided into 14 AOIs (e.g., LDLPFC and RDLPFC).

All preprocessed data of AOIs were entered into FC analysis. For each AOI, the HbO signal during each experimental condition were extracted, in which the time window was about 0–120 s after instruction onset of the block. Then, Pearson’s correlation coefficients (i.e., *r* values) were computed between all AOI pairs for each participant and for each experimental condition. Fisher’s *z* transformation was applied to convert *r* values to the normally distributed variable *z*. Next, the *z* values in the FT task were normalized to the M task, and those in the BT task were normalized to the E task, so that the values in each translation task were normalized to its own baseline. The normalized *z* values of each translation task were analyzed using one sample *t*-test for the IG and NIG, in order to explore the FC of sight translation in a single condition/group. The following analysis of the sight translation task, the two-way mixed design ANOVA, was conducted to compare the FC difference between the IG and NIG. The false discovery rate (FDR) correction was used correct for multiple comparisons (i.e., 91 possible pairs of AOIs). The alpha level of FDR correction 0.05.

## 3. Results

### 3.1. Behavioral Performance

**Figure 2:**
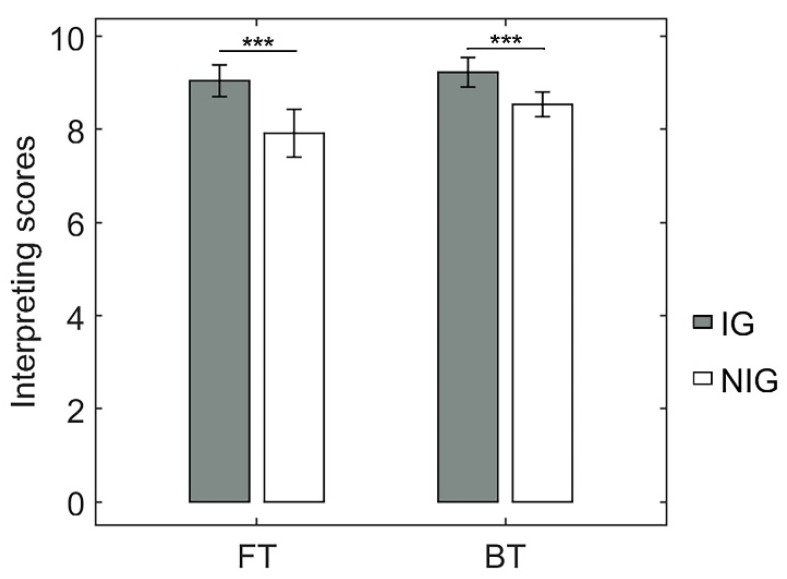
Behavioral performance of the translation tasks. *** *p* < 0.001.

To examine whether the IG in this study showed superiority, we compared the rating scores in sight translation tasks (i.e., BT and FT) from the NIG. The ANOVA showed a significant 2-way interaction (*F*_(1, 30)_ = 4.68, *p* = 0.04, *partial η*^2^ = 0.14). Further *t*-tests showed that the IG was significantly better than the NIG at the translation tasks, both at the FT and BT tasks (FT: *t* = 3.88, *p* < 0.001, Cohen’s *d* = 1.24; 9.05 ± 0.64 > 7.93 ± 0.97; BT: *t* = 3.54, *p* = 0.001, Cohen’s *d* = 0.77; 9.24 ± 0.61 > 8.55 ± 0.49; Figure 2). These results indicated that the IG was much more skilled at sight translation than the NIG regardless of translation direction.

Similar analysis on the reading tasks revealed a significant two-way interaction (*F*_(1, 30)_ = 18.15, *p* < 0.001, *partial η*^2^ = 0.38). Further *t*-tests showed that the IG performed equally as the NIG in reading Mandarin sentences (*t* = 0.69, *p* = 0.50, Cohen’s *d* = 0.05; 9.99 ± 0.02 ≈ 9.98 ± 0.03), but they were better than the NIG for reading English sentences (*t* = 4.30, *p* < 0.001, Cohen’s *d* = 2.08; 9.89 ± 0.08 > 9.61 ± 0.25). That is, both the IG and NIG had good oral English skills (i.e., pronunciation and fluency), but the IG was slightly better than the NIG.

### 3.2. Functional Connectivity in IG

**Figure 3:**
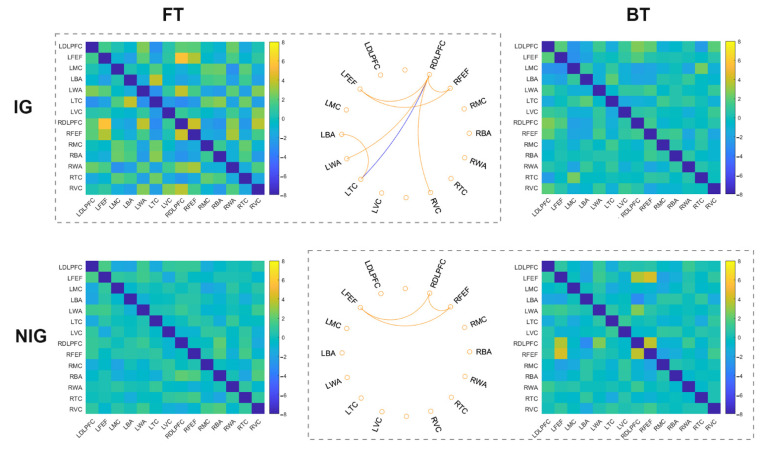
The results for each translation task for the IG and NIG. Circle plots showed significant connections after FDR correction (yellow: increased FC; blue: decreased FC). Heat maps displayed *t* values of each connection among the AOIs (DLPFC: dorsolateral prefrontal cortex; FEF: frontal eye fields; MC: motor cortex; BA: Broca’s area; WA: Wernicke’s area; TC: temporal cortex; VC: visual cortex; L: left; R: right).

The one-sample *t*-test revealed that in the FT task, the IG showed significant increased connections within the frontal cortex (e.g., left frontal eye fields–right frontal eye fields, right dorsolateral prefrontal cortex–left frontal eye fields, right dorsolateral prefrontal cortex–right frontal eye fields), between the frontal and visual/Wernicke’s areas (e.g., right dorsolateral prefrontal cortex–right visual cortex, right dorsolateral prefrontal cortex–left Wernicke’s area), and between left Broca’s area and left temporal cortex (*t*s > 3.31, corrected *p*s < 0.05, Cohen’s *d*s > 0.82) (Figure 3). There were significant decreased connections in right dorsolateral prefrontal cortex–left temporal cortex for the FT task (*t* = −3.16, corrected *p* < 0.05, Cohen’s *d* = 0.79). However, when performing the BT task, no significant FC was observed for the IG (−2.82 < *t*s < 2.65, corrected *p*s > 0.05). These results suggest that when performing sight translation from L1 to L2, the IG depend on the co-working within the frontal cortex, between the frontal, visual/Wernicke’s areas.

### 3.3. Functional Connectivity in NIG

In the NIG, the one-sample *t*-test revealed non-significant result for the FT task, either for the increased or decreased connection (−1.82 < *t*s < 2.01, corrected *p*s > 0.05). In the BT task, the NIG showed significant increased connections between right dorsolateral prefrontal cortex, right frontal eye fields, and left frontal eye fields (*t*s > 3.51, corrected *p*s < 0.05, Cohen’s *d*s > 0.87), while non-significant decreased connection (*t*s > −3.12, corrected *p*s > 0.05) (Figure 3). These findings indicate that the NIG rely on the functional connectivity within the frontal cortex for the translation from L2 to L1.

### 3.4. Functional Connectivity Difference between IG and NIG

**Figure 4:**
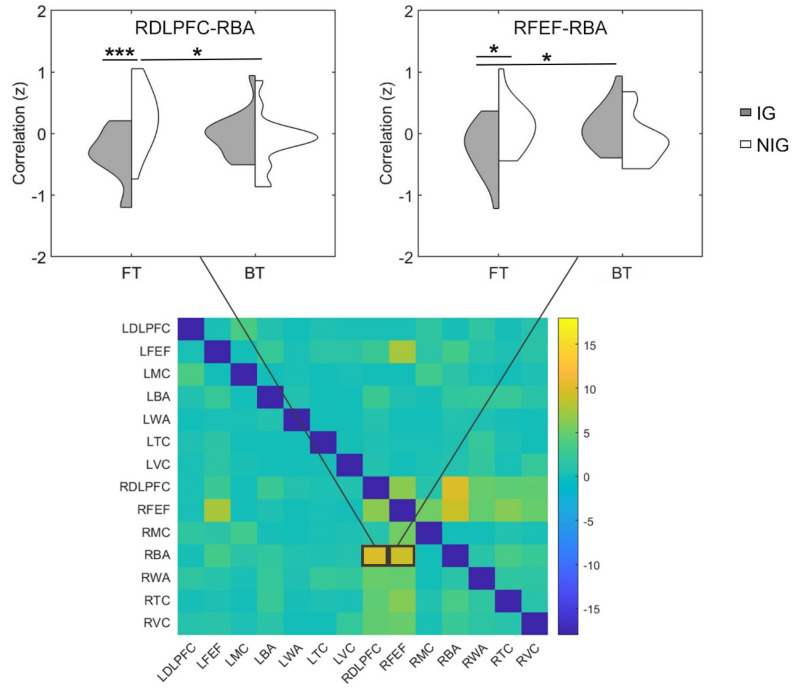
The results comparing the FC to the IG vs. NIG for the FT and BT task. Heat maps displayed *F* values of each connection among the AOIs (DLPFC: dorsolateral prefrontal cortex; FEF: frontal eye fields; MC: motor cortex; BA: Broca’s area; WA: Wernicke’s area; TC: temporal cortex; VC: visual cortex; L: left; R: right). * *p* < 0.05, *** *p* < 0.001.

The two-way (i.e., group × translation mode) ANOVA showed that the main effect of translation mode was not significant (*F*s < 4.90, corrected *p*s > 0.05, *partial η*^2^s < 0.14), which suggest that the FT and BT tasks equally recruits information exchange within the frontal cortex in all participants. The main effect of group was also non-significant in our AOIs (*F*s < 5.83, corrected *p*s > 0.05, *partial η*^2^s < 0.16). However, the interaction between group and translation mode was significant in right dorsolateral prefrontal cortex–right Broca’s area connection and right frontal eye fields–right Broca’s area connection (*F*s > 9.01, corrected *p*s < 0.05, *partial η*^2^s > 0.23; Figure 4). Further simple test showed that in the FT task, the two connections (i.e., right dorsolateral prefrontal cortex–right Broca’s area, right frontal eye fields–right Broca’s area) were both stronger in the NIG than the IG (*t*s > 2.47, *p*s < 0.05, Cohen’s *d*s > 0.88) (Figure 4). In addition, the NIG showed greater right dorsolateral prefrontal cortex–right Broca’s area connection for the FT task than the BT task (*t* = 2.49, *p* = 0.02, Cohen’s *d* = 0.88), while the IG exhibited higher right frontal eye fields–right Broca’s area connection for the BT task than the FT task (*t* = 2.58, *p* = 0.02, Cohen’s *d* = 0.91). These findings indicated that the IG and NIG depend on similar brain connections (e.g., the FC within the frontal cortex) when performing sight translation, but the two groups recruit different FC patterns (e.g., the IG depend less on the FC within the frontal cortex).

### 3.5. Associations between Functional Connectivity and Sight Translation Performance

The Pearson correlation analysis was conducted to examine the association between FC and sight translation performance in sight translation tasks. The analyses indicated no significant correlations between significant FC in our AOIs and rating scores in the translation tasks for each group (IG: *r*s < 0.49, *p*s > 0.05; NIG: *r*s < 0.20, *p*s > 0.45).

In consideration of the different experiences and proficiencies of the participants, their subjective reports were included as control variables. A partial correlation analysis showed that the correlation between the right dorsolateral prefrontal cortex–left frontal eye fields connection/ right dorsolateral prefrontal cortex–right visual cortex connection and rating scores in the FT task was significantly positive for the IG (*r*s > 0.57, *p*s <0.04). No other significant correlation was observed (IG: *r*s < 0.52, *p*s > 0.05; NIG: *r*s < 0.24, *p*s > 0.39).

## 4. Discussion

In the present study, we explored the FC underlying simultaneous translation tasks in interpreters and non-interpreters, by using the non-invasive fNIRS technique. Our AOIs included bilateral inferior gyrus and inferior parietal lobule, as well as the dorsolateral prefrontal cortex. The results shed light not only on FC during simultaneous language translation, but also reveal the relevance of fNIRS for a similar investigation. Future studies examining this issue are likely to provide equally interesting findings by utilizing other methodologies. We will summarize and discuss our findings in the following paragraphs.

Our findings revealed that both interpreter and non-interpreter groups recruited the right dorsolateral prefrontal hub when completing the sight translation task, indicating the paramount role it plays in simultaneous language translation. The dorsolateral prefrontal hub is suggested to be involved in multiple demand system, functioning in cognitive-attentional-executive control, language switching, as well as the management of non-literal meanings [61]. This result well supports previous studies that have identified the activation of the dorsolateral prefrontal region in simultaneous language translation [17,23,24,62], suggesting such cognitive processes as planning, inhibition, task monitoring, and language switching are involved when completing the task [34,35,36]. It is worth mentioning that most studies in the existing literature have identified the dorsolateral prefrontal hub in the left hemisphere [5,6]. Yet, we found the dorsolateral prefrontal hub in the right hemisphere was recruited in our experiment. The discrepancy might be credited to the language pairs involved in simultaneous translation tasks. In most previous work, the two languages involved for the bilingual participants were both alphabetic languages. In contrary, the processing of Mandarin, an ancient ideographic language, has been reported to be associated with different or additional brain areas [63,64]. The preoperative and intraoperative study conducted by Wang et al. [65] revealed the involvement of right hemisphere in Mandarin processing during auditory naming, free fluency, verbal fluency letter and verbal fluency characters tasks. Moreover, studies have suggested the recruitment of the right hemisphere, including the right dorsolateral prefrontal cortex, as well as the right Broca’s area in Mandarin language processing [8,66,67]. For instance, Liu et al. [66] concluded that the right dorsolateral prefrontal cortex might be responsible for inhibitory control in Mandarin/English language switching, concomitant with an altered electrophysiological late positive component. However, experimental studies addressing the role of the right dorsolateral prefrontal cortex in the cognitive processing of Mandarin are still rare, which might be an interesting area for future research.

Based on previous neuroimaging studies we hypothesized a different FC pattern during simultaneous translation between interpreters and non-interpreters. As expected, we found different FC results between two groups. The interpreter group had brain connection between the frontal and Wernicke’s area/visual cortex while the non-interpreter group between the frontal and left/right frontal eye fields. This incongruence of connectivity is consistent with the finding reported by Becker et al. [5] who also found different brain connection during non-translation tasks between simultaneous interpreters and multilingual controls. Studies have indicated neuroplastic changes in the frontal interconnected brain network due to multitasking training [5]. Our finding that the frontal area is connected to different language subregions in interpreters compared to that in non-interpreters during simultaneous language translation could also reflect the functional neuroplasticity following multitask training in simultaneous language translation.

FC between the frontal and Wernicke’s area was found in the interpreter group during forward sight translation. This result means that simultaneous language translation with interpreters is characterized by information exchange between Wernicke’s area and the frontal region. Studies [68,69] have found the frontal and temporoparietal areas constitute critical speech processing networks. FC between Broca’s area and Wernicke’s area during interactive verbal communication has been identified [68]. Wernicke’s area is suggested to play a role in short-term memory tasks and speech output [32]. The FC found in this group might indicate interpreters have developed a functional network that mediates attention, executive control, retrieval of mental representation, as well as speech production. We might further suggest the accumulative simultaneous language translation training across lifespan has influence on the temporal alignment of neural coupling between the frontal and Wernicke’s area in interpreters. This result partially aligns with García et al. [12] who found greater connectivity in bilateral frontotemporal networks in 10 professional translators during forward word translation. In comparison, the non-interpreter group revealed FC between the frontal cortex and Broca’s area during their backward sight translation. This result indicates the information exchange between these two regions in non-interpreters. Broca’s area, being part of the dorsal and ventral frontotemporal language network [61], is suggested to support sentence generation. The connectivity between the frontal cortex and the language areas (BA 44, BA45) plays a role in providing a continuous and coherent flow of speech and in maintaining error detection and repairment mechanisms, during speech production as well as perception [70]. The FC identified in this group might suggest non-interpreters relied heavily on this network to maintain, monitor and correct their sight translation output. This is consistent with the behavioral result that non-interpreters averagely scored significantly lower than interpreters regarding the performance.

Our findings also reveal that interpreters have more connectivity pathways (i.e., right dorsolateral prefrontal cortex-right visual cortex, right dorsolateral prefrontal cortex-Wernicke’s area, right dorsolateral prefrontal cortex-frontal eye fields) than non-interpreters, suggesting the information exchange in interpreters involves more brain regions. This might partially support the interpreter advantage hypothesis which argues that interpreters have more cognitive flexibility when completing tasks [44]. The multiple connectivity pathways found in interpreters may also well exemplify the “multiple demand system”, which, in particular the dorsolateral prefrontal cortex as its center, functions to link other networks in dependency of demands required by specific tasks [71]. In the present study, the right dorsolateral prefrontal hub was recruited by both groups during simultaneous language translation, yet it was functionally connected to different language subregions. This seems to suggest the cognitive control network is connected to different functional networks related to various language domains in an expertise-specific way.

In the present study, fNIRS, a facilitating tool for brain imaging, was used to map the FC underlying Mandarin/English simultaneous language translation with interpreters and non-interpreters. To the best of our knowledge, this study is the first that uses optical neuroimaging to explore the FC underlying Mandarin/English sight translation. As expected, our novel findings revealed that the FC pattern varies between interpreters and non-interpreters during simultaneous language translation. Specifically speaking, we found the FC between the frontal cortex and Wernicke’s area in interpreters. In contrast, non-interpreters are characterized by the FC between the frontal and Broca’s area. These findings reveal significant differences between the neural coupling mechanisms in interpreters and non-interpreters. However, the conclusions we draw are limited by the spatial and temporal resolution of the fNIRS technique, whose measurements are restricted to the cortical surface and whose signals are relatively slow compared to EEG and MEG. The findings could also be limited by the sample size in the present study; thus, follow-up studies should be conducted to further investigate this issue.

## Figures and Tables

**Table 1:** Characteristics of the study groups.

Item	IG (*n* = 16)		NIG (*n* = 16)	
	Mean	SD	Mean	SD
Age (years)	25.00	1.08	23.88	1.59
Education	Bachelor’s degree		Bachelor’s degree	
Working memory span amplitude (maximum)	2.75	0.45	2.88	0.72
L2 proficiency score (maximum: 6)	4.56	0.81	4.69	0.60
Age of first acquisition of L2 (years)	10.19	2.04	8.63	2.80
Total time length since L2 acquisition (years)	14.75	2.91	15.19	2.14

**Table 2:** Comparability of Mandarin and English sentences.

	Mandarin Sentences M (SD)	English Sentences M (SD)	*p*
Word count	10 (0.52)	10 (0.35)	1
Notional word density	78.95% (0.01)	78.09% (0.01)	0.71
Pronoun density	10.51% (0.00)	11.67% (0.00)	0.22
Translatability	3.76 (0.46)	3.80 (0.92)	0.86
Word frequency	Mandarin sentences M (SD)	English sentences M (SD)	*p*
1–1000	69.46% (0.01)	68.60% (0.01)	0.72
1001–2000	8.36% (0.00)	8.40% (0.00)	0.98
2001–3000	4.55% (0.00)	4.14% (0.00)	0.82
3001–4000	0.83% (0.00)	1.67% (0.00)	0.47
4001–5000	2.64% (0.00)	2.93% (0.00)	0.86
5000 plus	14.15% (0.01)	14.26% (0.01)	0.96

## Data Availability

The data presented in this study are available on request from the corresponding author.
